# Arthroscopic partial meniscectomy versus physical therapy for traumatic meniscal tears in a young study population: a randomised controlled trial

**DOI:** 10.1136/bjsports-2021-105059

**Published:** 2022-06-08

**Authors:** Sabine J A van der Graaff, Susanne M Eijgenraam, Duncan E Meuffels, Eline M van Es, Jan A N Verhaar, Dirk Jan Hofstee, Kiem Gie Auw Yang, Julia C A Noorduyn, Ewoud R A van Arkel, Igor C J B van den Brand, Rob P A Janssen, Wai-Yan Liu, Sita M A Bierma-Zeinstra, Max Reijman

**Affiliations:** 1 Orthopaedics and Sports Medicine, Erasmus MC University Medical Centre, Rotterdam, The Netherlands; 2 Orthopaedics, Noordwest Hospital Group, Alkmaar, The Netherlands; 3 Orthopaedics, Sint Antonius Hospital, Nieuwegein, The Netherlands; 4 Orthopaedic Surgery, Joint Research, OLVG, Amsterdam, The Netherlands; 5 Orthopaedics, Haaglanden Medical Centre, Den Haag, The Netherlands; 6 Orthopaedics, Elisabeth-TweeSteden Hospital, Tilburg, The Netherlands; 7 Orthopaedic Surgery, Maxima Medical Centre, Eindhoven, The Netherlands; 8 Department of Biomechanical Engineering, Eindhoven University of Technology, Eindhoven, The Netherlands; 9 Orthopaedic Surgery, Catharina Hospital, Eindhoven, The Netherlands; 10 Department of General Practice, Erasmus MC University Medical Center, Rotterdam, The Netherlands

**Keywords:** Meniscus, Randomized Controlled Trial

## Abstract

**Objective:**

To compare outcomes from arthroscopic partial meniscectomy versus physical therapy in young patients with traumatic meniscal tears.

**Methods:**

We conducted a multicentre, open-labelled, randomised controlled trial in patients aged 18–45 years, with a recent onset, traumatic, MRI-verified, isolated meniscal tear without knee osteoarthritis. Patients were randomised to arthroscopic partial meniscectomy or standardised physical therapy with an optional delayed arthroscopic partial meniscectomy after 3-month follow-up. The primary outcome was the International Knee Documentation Committee (IKDC) score (best 100, worst 0) at 24 months, which measures patients’ perception of symptoms, knee function and ability to participate in sports activities.

**Results:**

Between 2014 and 2018, 100 patients were included (mean age 35.1 (SD 8.1), 76% male, 34 competitive or elite athletes). Forty-nine were randomised to arthroscopic partial meniscectomy and 51 to physical therapy. In the physical therapy group, 21 patients (41%) received delayed arthroscopic partial meniscectomy during the follow-up period. In both groups, improvement in IKDC scores was clinically relevant during follow-up compared with baseline scores. At 24 months mean (95% CI) IKDC scores were 78 (71 to 84) out of 100 points in the arthroscopic partial meniscectomy group and 78 (71 to 84) in the physical therapy group with a between group difference of 0.1 (95% CI −7.6 to 7.7) points out of 100.

**Conclusions:**

In this trial involving young patients with isolated traumatic meniscal tears, early arthroscopic partial meniscectomy was not superior to a strategy of physical therapy with optional delayed arthroscopic partial meniscectomy at 24-month follow-up.

**Trial registration:**

https://www.trialregister.nl/trials.

**WHAT IS ALREADY KNOWN ON THIS TOPIC**

- Young patients with acute meniscal tears in previously healthy knees are usually offered arthroscopic surgery. There is a widespread belief that surgery is needed but no high-level trials have investigated arthroscopic partial meniscectomy compared with non-operative treatment.

**WHAT THIS STUDY ADDS**

- Young patients (aged 18–45 years) with traumatic meniscal tears who were treated with arthroscopic partial meniscectomy, compared with those who had physical therapy plus optional delayed arthroscopic partial meniscectomy, had similar perceptions of symptoms, knee function and ability to participate in sports at 24-month follow-up.
- Fifty-nine per cent of the patients randomised to physical therapy did not undergo delayed arthroscopic partial meniscectomy during the follow-up period.

**HOW THIS STUDY MIGHT AFFECT RESEARCH, PRACTICE AND/OR POLICY**

- Results from this study suggest that physical therapy with optional delayed arthroscopic partial meniscectomy is a reasonable alternative to early arthroscopic partial meniscectomy in patients with a traumatic meniscal tear.

## Introduction

Arthroscopic partial meniscectomy is the most frequently performed orthopaedic surgery in the world.^1–3^ Around 500 000 partial meniscectomies are performed annually in the USA, of which 40% in patients under 45 years.^3 4^ In middle age and older patients with chronic degenerative tears, multiple high level studies showed that partial meniscectomy has no benefit compared with non-operative treatment.^5–8^ These studies led to new clinical practice guidelines making a strong recommendation against arthroscopic treatment and recommending initial non-operative treatment for older patients with degenerative tears.^9 10^

Young patients with acute meniscal tears in previously healthy knees are usually offered surgery.^11 12^ There is a widespread belief that surgery is needed to diminish complaints such as locking and joint line pain but no high level trials have investigated arthroscopic partial meniscectomy compared with non-operative treatment.^12 13^ We conducted the first (to our knowledge) randomised controlled trial (RCT) in a young population (18 to 45 years) with traumatic meniscal tears in otherwise healthy knees, comparing the effectiveness of arthroscopic partial meniscectomy with physical therapy. The aim of our study was to investigate whether arthroscopic partial meniscectomy was superior to physical therapy in young patients with traumatic meniscal tears for IKDC score at 24-month follow-up.

## Methods

### Study design

The Study of Traumatic meniscal tears: Arthroscopic Resection vs Rehabilitation (STARR) trial was an open-labelled, multicentre, parallel RCT. The trial was designed as a superiority study. Patients were recruited between August 2014 and November 2018 in eight hospitals (one university hospital and seven non-university hospitals) in the Netherlands. The trial was registered in the Netherlands Trial Register prior to the inclusion of the first subject. Reporting follows the Consolidated Standards of Reporting Trials (CONSORT) guidelines.^14^

### Patient involvement

Our patient panel consisted of three patients with a traumatic meniscal tear. The trial setup was discussed with a panel of people with acute knee injuries before the subsidy request was submitted. In collaboration with these patients, we made our study protocol as similar as possible to our usual clinical follow-up periods and standard measurements. Since 2010, we have expanded our use of patient participation panels on a regular basis. We plan to disseminate the study results to study participants.

### Patients and enrolment

Patients were recruited from outpatient clinics of the participating hospitals, after referral to the outpatient clinic either by the accident emergency department or by the general practitioner. Patients aged 18–45 years with a knee trauma in the previous 6 months (a specific incident after which knee complaints started) and a grade 3 meniscal tear on MRI were eligible for study participation. A grade 3 meniscal tear has signal changes on MRI that reach the articular surface of the meniscus and therefore is considered to be a full tear.^15^ Exclusion criteria were: a locked knee (ie, when the patient was unable to fully extend or flex the injured knee, confirmed by clinical examination), a meniscal tear that was suitable for suture repair based on MRI findings,^16^ a concurrent rupture of the anterior or posterior cruciate ligament, radiographic signs of osteoarthritis in the index knee (Kellgren Lawrence^17^ grade 2 or higher), disabling comorbidity or insufficient command of the Dutch or English language. Patients could have minor cartilage damage, which was not visible on radiographs. Eligible patients received oral and standardised written trial information.

### Randomisation and allocation concealment

Following informed consent and baseline measurements, patients were randomised into one of the two treatment groups in a 1:1 ratio. Randomisation was stratified for participating orthopaedic surgeon. The enrolment personnel contacted one researcher (not otherwise associated with the trial) who allocated treatment arms using computer-generated random numbers (central randomisation). The type of randomisation was stratified balanced block randomisation. Treatment arms were allocated in block sizes varying from 2 to 6. During the interim analysis and the final analysis, the statistician was blinded for treatment allocation.

## Interventions

### Arthroscopic partial meniscectomy

Arthroscopy was scheduled within 6 weeks of randomisation. When the meniscal tear was considered not suitable for suture repair on baseline MRI, but turned out to be suitable for suture repair during the arthroscopy based on perioperative findings, the orthopaedic surgeon was allowed to suture the ruptured meniscus. All participating orthopaedic surgeons normally performed at least 50 knee arthroscopies annually. All costs of surgery were covered by patients’ health insurance. Postoperatively patients were treated according to routine clinical practice and the Dutch national guidelines, not all patients were actively referred to physical therapy but they were at liberty to do so.^18^

### Physical therapy

Patients were referred to a physical therapist for an individual standardised physical therapy programme lasting at least 3 months. This exercise programme was developed by an expert panel consisting of experienced orthopaedic surgeons, sport physicians and physical therapists, based on clinical practice and available evidence.^18 19^ The programme consisted of three phases: (I) reducing knee effusion; (II) optimising range of motion (a) and restoring coordination and muscle function (b); (III) stimulating activities in daily living and return to sport. See online supplemental appendix 1 for a detailed description of the exercise programme. The exercises were tailored to the individual. The frequency of physical therapy sessions was determined by the physical therapist, depending on the functional level of the patient and the patients’ knee status. Patients’ progress and compliance was actively monitored by the investigator and therapist. Patients also received a home exercise programme (online supplemental appendix 1). Pain was handled using regular pain medication, starting with paracetamol, supplemented with non-steroid anti-inflammatory drugs if necessary. All costs of physical therapy were covered; financial arrangements in this context were established with health insurance companies. After the 3 months of physical therapy if they had persistent knee complaints, patients could opt for surgery, in consultation with the orthopaedic surgeon.

10.1136/bjsports-2021-105059.supp1 Supplementary data

### Outcome measures

The primary outcome was the IKDC score after 24-month follow-up. The IKDC score measures the patient’s perception of symptoms, knee function and ability to participate in sports activities. IKDC score ranges from 0 to 100, where 100 is the optimal score. It is a widely used and validated patient reported outcome measure to evaluate the recovery of patients with meniscal injuries.^20^

Secondary outcomes were the Knee Injury and Osteoarthritis Outcome Score (KOOS), knee pain in rest and during activity (Numeric Rating Scale (NRS)), Lysholm, Western Ontario Meniscal Evaluation Tool (WOMET), sporting activity level (Tegner score) and satisfaction with knee function. KOOS consists of five subscales: pain, symptoms, activities of daily living, sports and quality of life (QoL) and ranges from 0 to 100, with 100 being the optimal score.^21^ NRS-pain ranged from 0 to 10, where 0 represented no pain. Lysholm ranges from 0 to 100, with 100 being the optimal score.^22^ WOMET ranges from 0 to 100, where 100 is the optimal score.^23^ WOMET is validated and reliable for assessing health related quality of life in patients with meniscal pathology.^24^ The Tegner score ranges from 0 to 10, with 10 being the highest activity score.^22^ Satisfaction ranged from 0 to 100, with 100 representing optimal satisfaction (visual analogue scale). Other secondary outcomes were serious adverse events (SAEs) (complications and reinterventions), which were recorded during patient visits to the outpatient clinic and retrieved from the patient records.^25^

Patients were seen at the outpatient clinic of the participating hospital at baseline and 12 and 24 months after randomisation. Patients completed all questionnaires digitally at 0, 3, 6, 9, 12 and 24 months, except for the KOOS and Lysholm questionnaire. The KOOS questionnaire was filled in at 0 and 24 months, the Lysholm questionnaire was filled in at 0, 12 and 24 months. Study data were collected and managed using GemsTracker electronic data capture tools hosted at Erasmus MC.^26^

When we calculated the sample size, no studies on minimal clinically important difference (MCID) for the IKDC score were available. We based our initial sample size calculation on detection of a difference with an effect size of 0.5 in favour of the arthroscopic partial meniscectomy group compared with the physical therapy group, with 80% power and a two-sided type I error of 5%. To allow for a potential loss to follow-up of 15% in 2 years and to compensate for per-operative conversions from meniscectomy to meniscal repair (estimated 5% in the arthroscopy group), the target sample was set to 158 patients (79 arthroscopic partial meniscectomy, 79 physical therapy).

During a planned interim report for the grant supplier, we had a lower loss to follow-up rate than anticipated. In the meantime, a MCID for the IKDC score of 13.9 in knee injury patients had been published.^27^ Based on a SD of 16.2 for this score at baseline in our study population so far, and based on the feasibility of recruitment in a reasonable time period, we agreed with the grant supplier on adjusting the sample size to 100 patients (50 arthroscopic partial meniscectomy, 50 physical therapy). As even with a much higher SD of 22, we still could detect a difference of 13.9 points with the same amount of loss to follow-up as initially anticipated. We reported the alteration in sample size in the trial register.

### Statistical analysis

In the primary analysis, patients were analysed according to their randomisation group. To answer our primary research question, we used a linear regression model with IKDC score after 2 years as dependent variable, adjusted for baseline IKDC, randomisation and surgeon. We checked the following model assumptions: linearity, multicollinearity, homoscedasticity and normality and independence of residuals in the linear regression model. None of the assumptions were violated. To estimate the IKDC scores at baseline, 3, 6, 9 and 12 months, we used a linear mixed model to evaluate the between group difference in IKDC score, as indicated by the interaction between time point and randomised allocation. IKDC scores at baseline, 3, 6, 9, 12 and 24-month follow-up were used as dependent variable. Randomised allocation, follow-up period and interaction between randomised allocation and follow-up period (multiplication of randomisation and follow-up period as interaction term) were added to the model as fixed factors. Orthopaedic surgeon, used as stratum in the randomisation procedure, was added into the model as random factor. The covariance structure was modelled as unstructured. The model was estimated using the restricted maximum likelihood. We checked the following model assumptions: linearity, homoscedasticity and normality of residuals. None of the model assumptions were violated. In the secondary analysis, we analysed between group difference at 24 months in KOOS, NRS-pain, Lysholm, WOMET, satisfaction with knee function and Tegner, by using a linear mixed model as reported for the primary analysis. In all analyses, statistical significance was set at the two-sided 0.05 level.

## Results

### Patients

**Figure 1:**
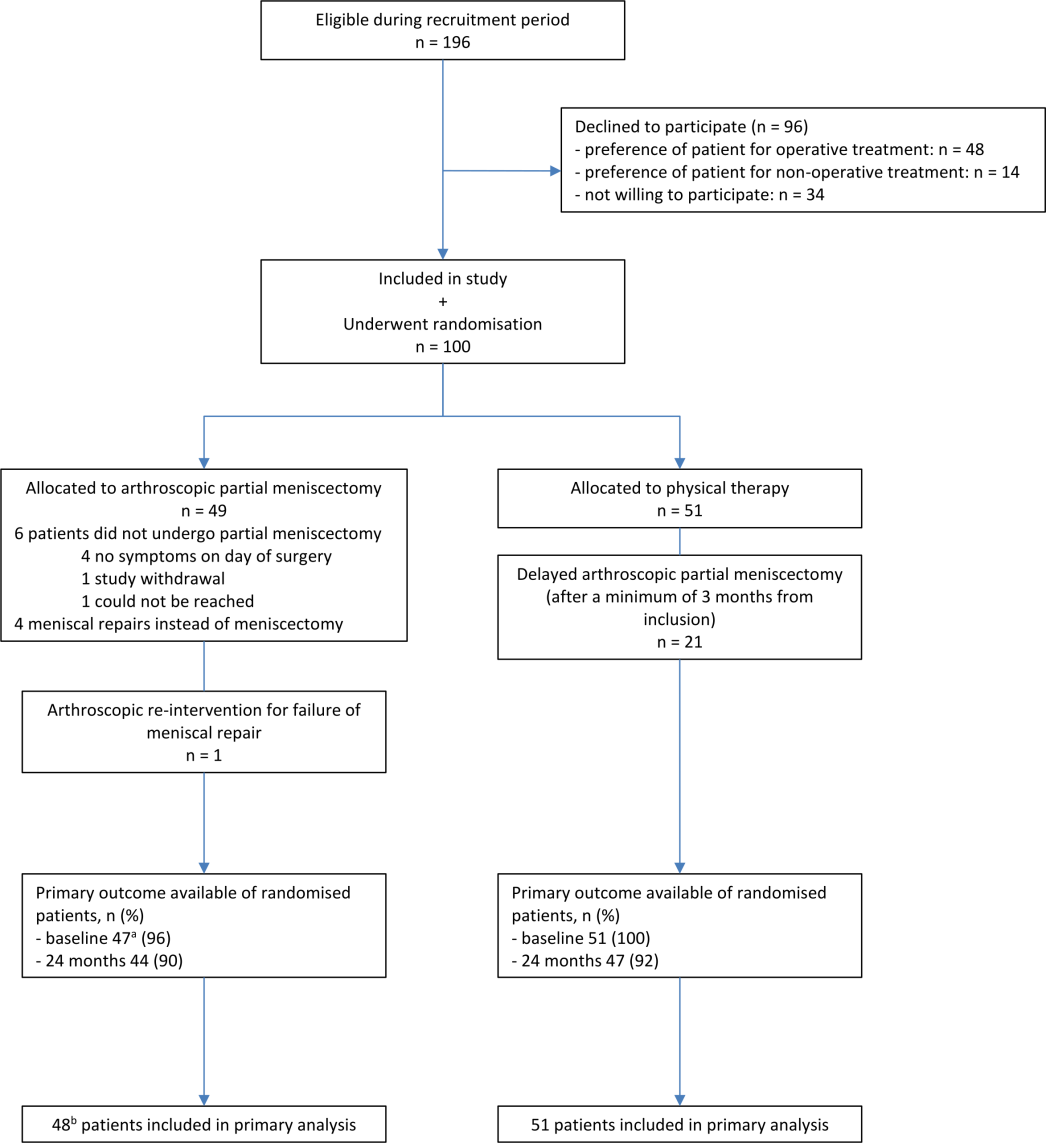
Flowchart. (A) minus 2 baseline questionnaires, 1 because of study withdrawal and 1 was incomplete. (B) minus 1 study withdrawal, who had no available data.

During the study period, 100 patients were included of the 196 who were eligible. Forty-nine patients were randomised to arthroscopic partial meniscectomy and 51 patients to physical therapy (see figure 1 and table 1). Final follow-up was completed for 91% of all included patients. Our study population included 34 competitive or elite athletes with a Tegner score of 8 or higher.

**Table 1:** Baseline characteristics

	Arthroscopic partial meniscectomy (n=48)	Physical therapy (n=51)
Age at inclusion, years	34.1 (8.6)	35.6 (7.5)
Male sex, n (%)	37 (77)	38 (75)
BMI (kg/m^2^)	25.5 (4.2)	26.1 (4.6)
Tegner preinjury*	6.5 (2.2)	6.4 (2.0)
Time between trauma and inclusion, days (median (IQR))	88 (48–150)	91 (58–149)
IKDC score†	46 (16)	47 (18)
KOOS score‡		
Pain	54 (20)	60 (21)
Symptoms	56 (20)	63 (18)
ADL	61 (22)	69 (22)
Sport	30 (25)	35 (29)
QoL	34 (18)	36 (18)
NRS-pain rest§	3.9 (2.5)	2.9 (2.8)
NRS-pain activity§	6.6 (2.4)	6.2 (2.4)
Lysholm score¶	67 (18)	70 (18)
WOMET score**	38 (18)	43 (19)
Meniscus injured during, n (%)		
Sport	27 (56)	27 (53)
Daily activities	5 (10)	11 (22)
Work	10 (21)	8 (16)
Other	5 (10)	5 (10)
Meniscal tear baseline MRI, n (%)		
Medial meniscus	31 (65)	35 (69)
Lateral meniscus	16 (33)	14 (27)
Medial+lateral meniscus	1 (2)	2 (4)

Data are presented as mean with SD in brackets unless otherwise reported.

Some values of the arthroscopic partial meniscectomy group are known for 47 patients instead of 48.

*The Tegner scores ranges from 0 to 10, with higher scores indicating a higher activity level.

†The IKDC score ranges from 0 to 100, with higher scores indicating less symptoms and a higher patient’s perception of knee function and ability to participate in sports activities.

‡The Knee Osteoarthritis Outcome Score ranges from 0 to 100, with higher scores indicating less pain and knee symptoms, less problems with ADL and sport and a better QoL.

§The NRS for pain ranges from 0 to 10, with higher scores indicating more pain.

¶The Lysholm score ranges from 0 to 100, with higher scores indicating less knee symptoms and higher levels of functioning.

**The WOMET normalised score ranges from 0 to 100, with higher scores indicating a higher health-related quality of life.

ADL, activities of daily living; IKDC, International Knee Documentation Committee; KOOS, Knee Injury and Osteoarthritis Outcome Score; NRS, Numeric Rating Scale; QoL, quality of life; WOMET, Western Ontario Meniscal Evaluation Tool.

Six patients (12%) of the arthroscopic partial meniscectomy group received no surgical treatment; in four patients’ complaints had resolved before surgery, one patient withdrew from the study and one patient could not be reached. In four patients (8%) in the surgical group, the surgeon decided during surgery to perform meniscal repair instead of partial meniscectomy, based on arthroscopic findings. Twenty patients in the surgery group (42%) had one or more physical therapy sessions in the first 3 months after inclusion, median of 5.0 sessions, IQR 2.0–8.0.

In the physical therapy group, the median number of physical therapy sessions was 8.5 per patient (IQR 4.0–12.0). Twenty-one patients (41%) of the physical therapy group underwent a delayed arthroscopic partial meniscectomy during the follow-up period in consultation with the orthopaedic surgeon, because of persistent complaints. The time between randomisation and delayed arthroscopic partial meniscectomy ranged from 3 to 21 months with a median duration of 5.5 months.

### Primary outcome

**Figure 2:**
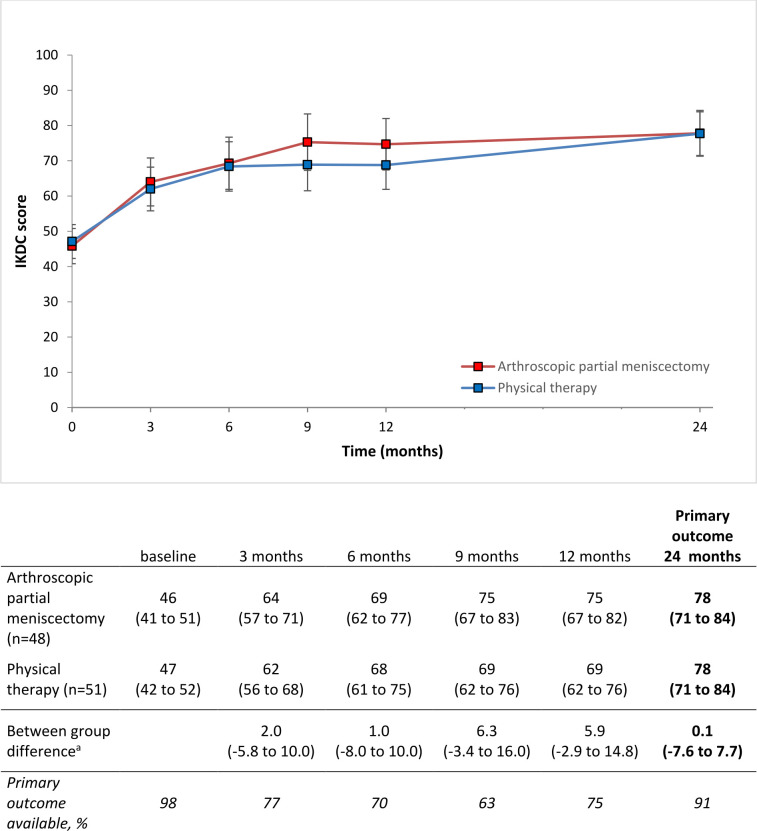
Estimated IKDC score^a^ for as randomised analyses per measurement period. (A) 3, 6, 9 and 12 months: adjusted for surgeon, 24 months: adjusted for baseline IKDC, randomisation and surgeon. Error bars represent 95% CI. Table: 95% CI in brackets. IKDC score ranges from 0 to 100, with higher scores indicating less symptoms and a higher patient’s perception of knee function and ability to participate in sports activities. IKDC, International Knee Documentation Committee.

We did not find that arthroscopic partial meniscectomy is superior to physical therapy in IKDC score at final follow-up of 24 months (between group difference 0.1; 95% CI −7.6 to 7.7; p value 0.99). Both groups improved in IKDC score during the 24-month follow-up period (figure 2). The change in IKDC score over the follow-up period and the between group differences during the different time points are shown in figure 2.

### Secondary outcomes

We did not find that arthroscopic partial meniscectomy was superior to physical therapy at 24 months in KOOS, NRS-pain, Lysholm, WOMET, Tegner and satisfaction with knee function (see table 2). All data for the secondary outcomes at each time point are in the online supplemental appendix 1.

**Table 2:** Secondary outcomes* for as randomised analyses 24-month follow-up

	Arthroscopic partial meniscectomy n=48	Physical therapy n=51	Between group difference
KOOS†			
Pain	86 (79 to 92)	84 (77 to 90)	1.9 (−5.7 to 9.6)
Symptoms	82 (75 to 88)	81 (75 to 88)	0.5 (−6.6 to 7.5)
ADL	92 (87 to 98)	89 (84 to 94)	2.8 (−3.3 to 8.9)
Sport	70 (61 to 80)	69 (60 to 79)	0.8 (−12.5 to 14.0)
QoL	67 (59 to 75)	66 (58 to 74)	1.4 (−9.3 to 12.0)
NRS-pain rest‡	1.2 (0.4 to 1.9)	1.2 (0.5 to 2.0)	−0.1 (−0.8 to 0.7)
NRS-pain activity‡	2.8 (1.9 to 3.7)	2.4 (1.5 to 3.3)	0.4 (−0.8 to 1.5)
Lysholm§	89 (85 to 94)	88 (84 to 93)	−1.0 (−6.2 to 4.1)
WOMET¶	72 (64 to 80)	76 (68 to 84)	−3.8 (−13.8 to 6.2)
Tegner**	5.4 (4.7 to 6.1)	5.0 (4.4 to 5.7)	0.3 (−0.6 to 1.3)
Satisfaction with knee function††	72 (64 to 80)	70 (62 to 78)	1.5 (−9.3 to 12.3)

Data are presented as adjusted mean estimate with 95% CI in brackets.

*Adjusted for surgeon.

†The Knee Osteoarthritis Outcome Score ranges from 0 to 100, with higher scores indicating less pain and knee symptoms, less problems with ADL and sport and a better QoL.

‡The NRS for pain ranges from 0 to 10, with higher scores indicating more pain.

§The Lysholm score ranges from 0 to 100, with higher scores indicating less knee symptoms and higher levels of functioning.

¶The WOMET normalised score ranges from 0 to 100, with higher scores indicating a higher health-related quality of life.

**The Tegner scores ranges from 0 to 10, with higher scores indicating a higher activity level.

††Satisfaction with knee function is measured using a visual analogue scale ranging from 0 to 100, with higher scores indicating a higher patients’ satisfaction with their knee function.

ADL, activities of daily living; NRS, Numeric Rating Scale; QoL, quality of life; WOMET, Western Ontario Meniscal Evaluation Tool.

### Serious adverse events

The number of SAEs is presented in table 3. In both groups, one patient underwent an arthroscopic intervention for a meniscal tear in the contralateral knee. In the arthroscopic partial meniscectomy group, one patient underwent an additional arthroscopic intervention because of failure of the meniscal repair. In the patients that underwent delayed arthroscopic partial meniscectomy, one patient was found to have an anterior cruciate ligament rupture, which was discovered during arthroscopy and was not visible on the baseline MRI. This anterior cruciate ligament rupture was reconstructed at 13-month follow-up.

**Table 3:** Serious adverse events

	Arthroscopic partial meniscectomy (n=48)	Physical therapy (n=51)
Arthroscopic intervention for meniscal tear in contralateral knee	1	1
Rupture of ACL with ACL reconstruction	0	1
Arthroscopic intervention for failure of meniscal repair	1	0
Non-knee related surgery or hospital admission	4*	2†

*1 surgery for carpal tunnel syndrome, 1 laparoscopy for abdominal cyst, 1 neurosurgery for brain tumour, 1 surgery for obstructive sleep apnoea syndrome

†1 surgery at the otorhinolaryngology department, 1 allergic reaction after intravenous contrast for contrast MRI for a femoral lesion

ACL, anterior cruciate ligament.

### osthoc analysis

The results of the posthoc as treated evaluations of the course in IKDC score are reported in online supplemental appendix 1. Four groups are reported: meniscal surgery, physical therapy, physical therapy plus delayed arthroscopic partial meniscectomy and no therapy (patients randomised to surgery that did not have surgery).

## Discussion

This is the first study to our knowledge comparing arthroscopic partial meniscectomy with physical therapy for traumatic meniscal tears in young patients with stable non-osteoarthritic knees. We did not find that arthroscopic partial meniscectomy was superior to physical therapy plus optional delayed arthroscopic partial meniscectomy at 24-month follow-up. Both groups showed clinically relevant improvements during the 24-month follow-up but did not achieve maximum IKDC scores at final follow-up. Fifty-nine per cent of the patients in the physical therapy group did not receive a delayed arthroscopic partial meniscectomy.

Arthroscopic surgery for meniscal injuries has become the most widely performed orthopaedic surgery in the world.^1–3^ This growth was based on a number of assumptions about the ability of surgery to achieve superior outcomes compared with non-surgical treatments. Over time, these have been questioned based on high-quality clinical studies. These high-quality studies examined the effectiveness in older patients with degenerative meniscal tears.^6 7^ This has led to a change in clinical practice guidelines. In the current guidelines, a strong recommendation is made against surgery and non-surgical treatment is recommended.^9 10^ Until recently, there were no clinical guidelines for traumatic meniscal injury. In 2019, the European Society for Sports Traumatology, Knee Surgery and Arthroscopy (ESSKA) published a consensus on the treatment of traumatic meniscal tears, stating that meniscus preservation should be the first choice of treatment.^12^ This consensus was based on low-quality evidence due to a lack of randomised studies. Clinical trials focusing on patients with traumatic meniscal tears are sparse, and RCTs comparing arthroscopic partial meniscectomy to non-operative treatment for this specific patient group were lacking. We studied a young homogeneous population with stable non-osteoarthritic knees and a clear isolated recent traumatic meniscal tear.

This study was designed as a superiority trial, and we did not find that arthroscopic partial meniscectomy was superior to physical therapy plus optional delayed arthroscopic partial meniscectomy in the treatment of traumatic meniscal tears. At 24-month follow-up, we found a between group difference of 0.1 out of 100 points in the IKDC scores of both treatment groups, with 95% CI of −7.6 to 7.7. To date, no papers have been published on the MCID of the IKDC score in traumatic meniscal injuries, but new data are now available on the MCID of the IKDC score in anterior cruciate ligament ruptures (13.9) and in degenerative meniscal injuries (10.9).^27 28^ Our 95% CI did not exceed both available MCIDs, neither did it exceed an effect size of 0.5 as used in the initial sample size calculation. Therefore, our study also showed that it is unlikely that arthroscopic partial meniscectomy is clinically relevant superior to physical therapy.

A strength of our study is that it is the first RCT investigating treatment of traumatic meniscal tears with a 24-month follow-up in a young study population. We had less loss to follow-up than expected.

Our study has several limitations. First, preference of patients for a treatment may have induced recruitment bias, and our results may therefore not apply to those with strong treatment preference. Second, the primary analysis is subject to selection bias due to missing data and absence of blinding patients for the intervention. Third, in the Dutch healthcare system, patients with knee complaints are mainly referred to an orthopaedic surgeon within several months after the trauma. We included patients with a wide range of time from trauma to inclusion, 0–6 months. This may have resulted in a subgroup of patients that already followed non-operative treatment before inclusion, which may have led to a better knee function at study enrolment. Given the comparable IKDC scores at baseline, these influences were equally divided between both treatment groups. In both groups, a similar number of patients reported that they had already received physical therapy before inclusion.

Although this is the first RCT in this context, our results suggest that there is a reasonable alternative to early arthroscopic partial meniscectomy as first-line treatment in patients with a traumatic meniscal tear. The challenge is predicting which patients will benefit from arthroscopic partial meniscectomy and who will improve with non-surgical treatment. Further studies should investigate whether we can already predict at an early stage who will need surgery and who will have good prognosis with physical therapy. In our study, 41% of the patients randomised to physical therapy still underwent an arthroscopic partial meniscectomy during follow-up. In studies investigating degenerative tears, 20%–30% of the patients who started with physical therapy crossed-over to arthroscopic partial meniscectomy.^5 6 8 29 30^ Changing the treatment paradigm for traumatic meniscal tears to a more conservative treatment may also have major impact on treatment costs and result in large healthcare savings.

## Conclusion

We did not find that arthroscopic partial meniscectomy was superior to physical therapy plus optional delayed arthroscopic partial meniscectomy at 24-month follow-up in young patients with isolated traumatic meniscal tears. Fifty-nine per cent of patients randomised to physical therapy did not undergo delayed arthroscopic partial meniscectomy during the follow-up period.

## Data Availability

Data are available on reasonable request. Individual de-identified participant data that underlie the results reported in this paper (text, tables, figures and appendices) and the study protocol will be shared if requested. Data will be available beginning 12 months and ending 5 years following publication of this paper. Data will be available for researchers who provide a methodologically sound scientific proposal, which has been approved by an ethical committee. Proof of the latter should be provided. Analyses should achieve the aims as reported in the approved proposal. Proposals for data should be directed to m.reijman@erasmusmc.nl.
