# Supplementary material for: Arthroscopic partial meniscectomy versus physical therapy for traumatic meniscal tears in a young study population: a randomised controlled trial
Source: Br J Sports Med. 2022 Jun 8;56(15):870–6. doi: 10.1136/bjsports-2021-105059 (PMC9304087; doi:10.1136/bjsports-2021-105059)
Supplement: Supplementary data [file bjsports-2021-105059supp001.pdf]

Erasmus MC
University Medical Center Rotterdam

Supplementary appendix

STARR-trial Physical Therapy Protocol……………………………………………………..2
Home exercises for meniscal tear……………………………………………………………3
Supplementary Figure 1...…………………………………………………………………11
Supplementary Table 1……………………………………………………………………12

Erasmus MC
University Medical Center Rotterdam

STARR-trial Physical Therapy Protocol

Phase	Goal	Activities
**I**	Reduce knee effusion	Explanation and education about meniscal injury; advice for daily activities and to stay in 'pain free range of motion'
		Exercises (partial weight bearing) within 'pain free range of motion', e.g. walking, cross-training, cycling
**IIa**	Optimize range of motion	Transfers: sit and to stand<br>Cycling<br>Optional: stair walking (patient dependent)
		Homework:<br>Extension and flexion<br>-Straighten and bend the knee<br>Practicing simple daily activities<br>-Squat, step up, pelvic bridge
**IIb**	Optimize coordination and muscle function	To maintain / improve gait<br>-Active dynamic gait<br>To improve muscle function of the quadriceps<br>To train proprioception
		Homework:<br>Pursue full (passive) extension<br>Practicing simple daily activities<br>-Squat, step up, pelvic bridge
**III**	Stimulate activities in daily living and return to sport	Dependent on patients preferences / background / work situation: daily life or sport specific exercises
		<u>Daily life-specific exercises :</u><br>Walking and turning<br>Kneeling, squatting, lifting<br>Practicing complex, multiple transfers<br>Practicing complex daily activities (e.g. turn + reach)
		<u>Sport-specific exercises :</u><br>Extended gait training (goal: increase of intensity), e.g. dribbling – skippings<br>Jumping
		Homework:<br>Practicing complex, multiple transfers<br>Practicing complex daily activities (e.g. turn + reach)

van der Graaff SJA, *et al. Br J Sports Med* 2022; 56:870–876. doi: 10.1136/bjsports-2021-105059

Erasmus MC
University Medical Center Rotterdam

Home exercises for meniscal tear

Ask your physical therapist for advice and support

Erasmus MC
University Medical Center Rotterdam

Walking on a treadmill

- Start with walking
- Hold the rails if necessary
- Ask your physical therapists for advice on speed and technique

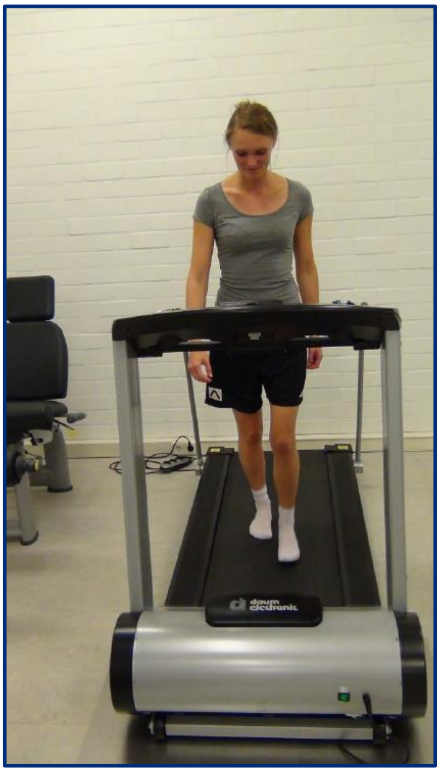

Erasmus MC
University Medical Center Rotterdam

Cycling on an exercise bike

- Adjust the bike to achieve a comfortable position
- Ask your physical therapist for advice on the cycling speed and changing the saddle height to increase bending of your knee

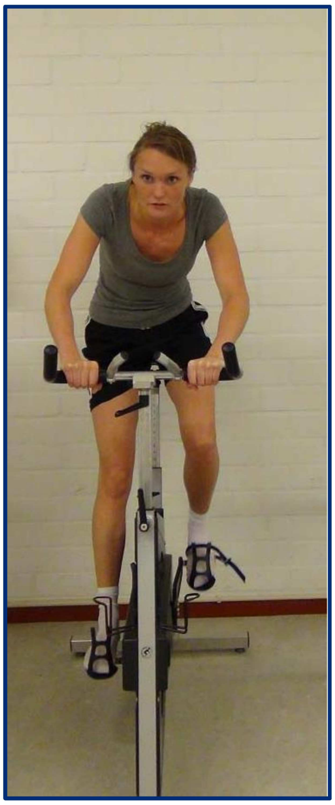

Erasmus MC
University Medical Center Rotterdam

Get up from a chair (squat)

- Use a chair on a flat floor
- Sit down with a straight back with your knees in a 90 degrees angle
- Put your arms straight out (figure 1)
- Keep your knees pointing forwards, avoid a knocked knee position (figure 3)
- Stand up, while keeping your knees and arms pointing forwards, until your legs are straight (figure 2)
- Repeat this exercise 15 times, two to three times a day
- When you succeed in this exercise, adjust the exercise by placing the foot of your injured leg slightly backwards (see figure 4) and perform the exercise in the same way

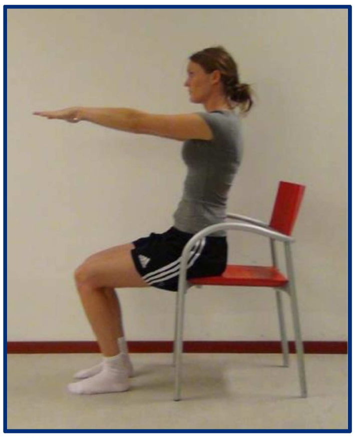

Figure 1

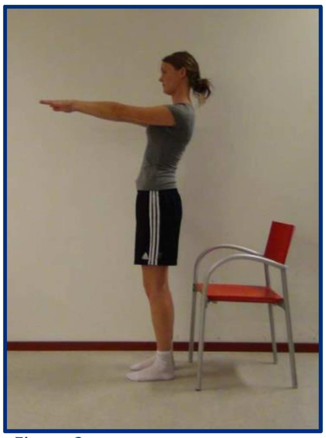

Figure 2

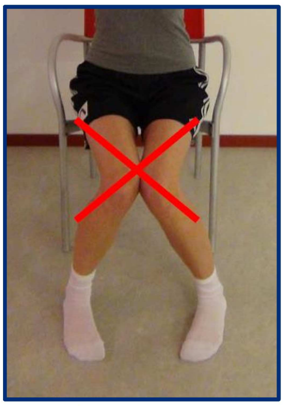

Figure 3

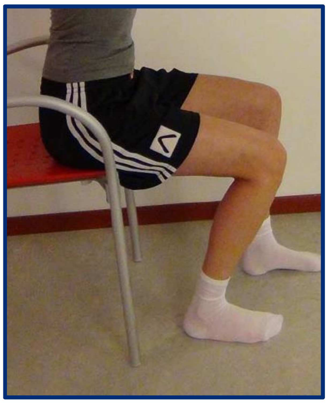

Figure 4

Erasmus MC
University Medical Center Rotterdam

Straighten the leg (extension)

- Use a bench approximately 40 centimeters high
- Place your hands on the bench, shoulder width apart, with your knees bent
- Elevate your uninjured leg (figure 1) while keeping your hands on the bench
- Pay attention to your knees, keep them pointing forwards, don't let your knees knock (figure 5)
- Straighten the injured leg (standing leg), while keeping your hands on the bench (figure 2)
- Repeat this exercise 10 times, two to three times a day
- When you succeed in this exercise, adjust the exercise by using a lower bench (20 centimeters) and perform the exercise in the same way (figure 3 and 4)

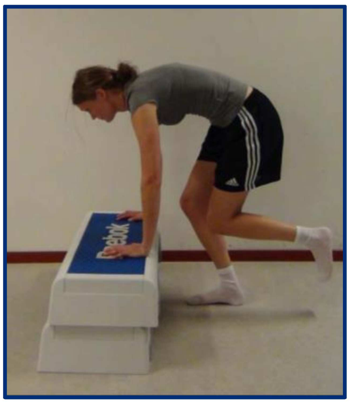

Figure 1

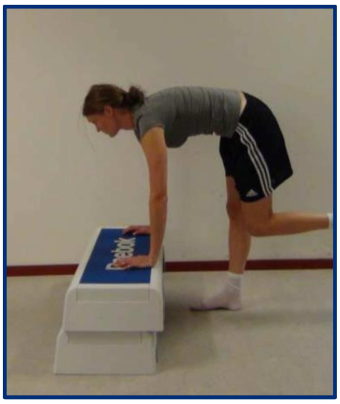

Figure 2

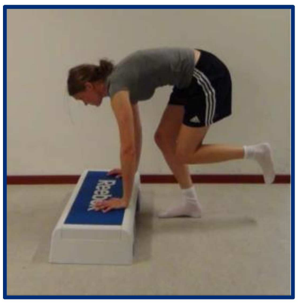

Figure 3

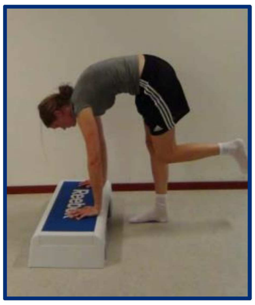

Figure 4

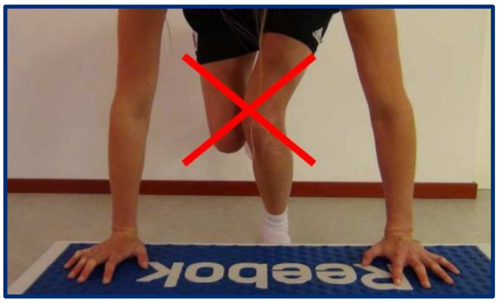

Figure 5

van der Graaff SJA, *et al. Br J Sports Med* 2022; 56:870–876. doi: 10.1136/bjsports-2021-105059

Erasmus MC
University Medical Center Rotterdam

Step-up

- Use a bench or chair at knee-height (if this is too hard, use a lower bench or chair)
- Stand in front of the bench
- Keep your uninjured leg straight on the floor and place your injured leg on the bench while bending your injured knee (figure 1)
- Step up onto the bench, by straightening your injured leg and elevating your uninjured leg to 90 degrees. Pay attention to your injured leg, it has to be straightened completely.
- Pay attention to the knee of your injured leg, it has to point forwards, not go into a knocked knee position (figure 3)
- Keep your back straight and keep looking forwards (figure 4)
- Repeat this exercise 10 times, two to three times a day

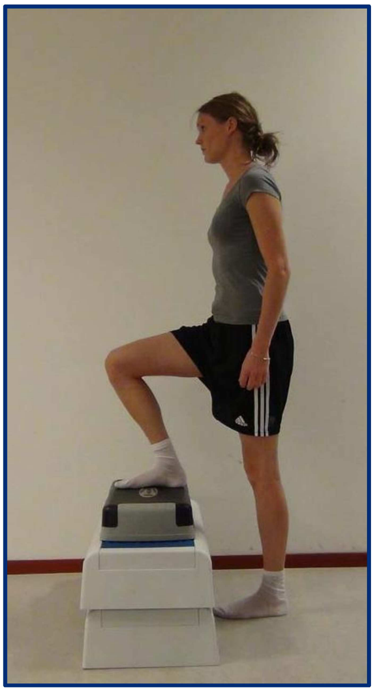

Figure 1

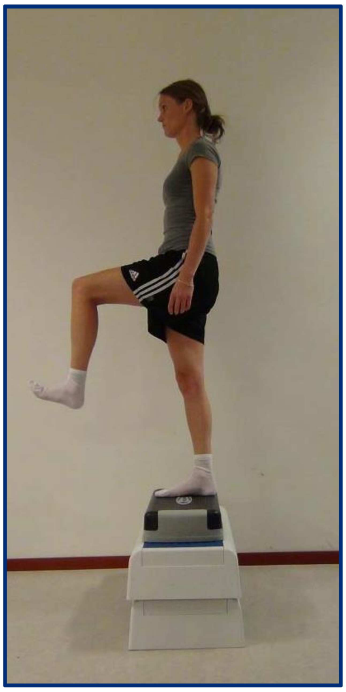

Figure 2

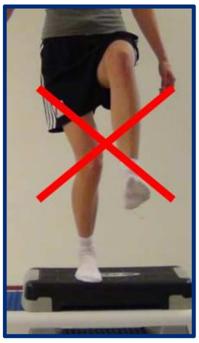

Figure 3

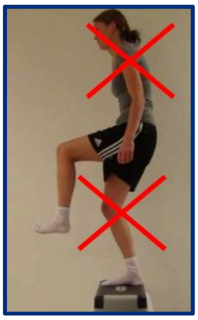

Figure 4

BMJ Publishing Group Limited (BMJ) disclaims all liability and responsibility arising from any reliance placed on this supplemental material which has been supplied by the author(s)

Erasmus MC
University Medical Center Rotterdam

Pelvic bridge

- Lay down with your arms at your sides
- Bend your knees to an angle of 110 degrees (figure 1)
- Keep your feet flat on the floor
- Keep your head on the floor
- Lift your pelvis, while keeping your feet, arms and head on the floor (figure 2)
- Keep your upper legs in a straight line with your belly
- Hold this position for 5 seconds
- Repeat this exercise 15 times, two to three times a day, for three weeks in a row
- After 3 weeks and when you succeed in this exercise, you can adjust the exercise by straightening the injured leg and placing your arms in front of you (figure 3). Repeat this exercise 15 times, two or three times a day

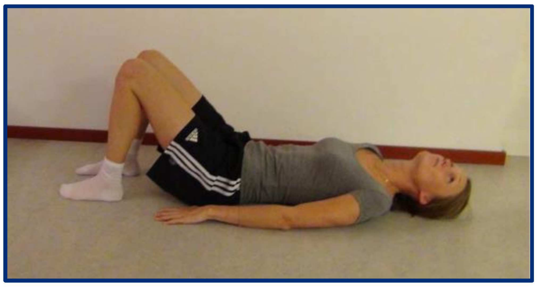

Figure 1

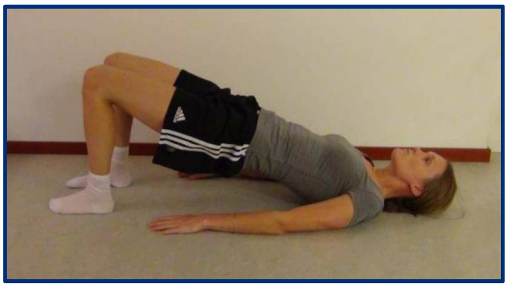

Figure 2

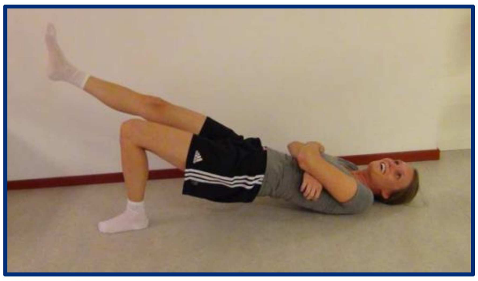

Figure 3

Erasmus MC
University Medical Center Rotterdam

Turn and reach

- Use a weight or water bottle of 0.5 to 1 kilograms
- Stand with your legs hip-width apart and place the weight on the side of the injured leg
- Move your uninjured leg back, with your toes still touching the floor (figure 1)
- Reach down with your arm from the uninjured side to the weight, while slightly bending your injured leg (figure 2)
- Grab the weight and straighten your injured leg, while keeping your uninjured leg with the toes on the floor (figure 3) until you are in the starting position again
- Repeat this exercise 10 times, two to three times a day
- When you can easily do in this exercise, make it harder by lifting your uninjured leg of the floor during the exercise (figure 4, 5 and 6)

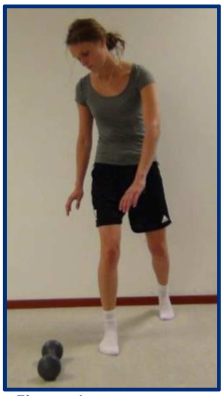

Figure 1

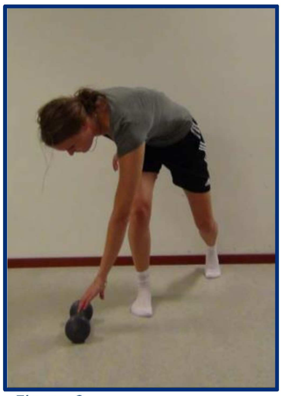

Figure 2

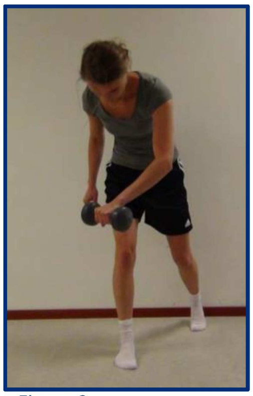

Figure 3

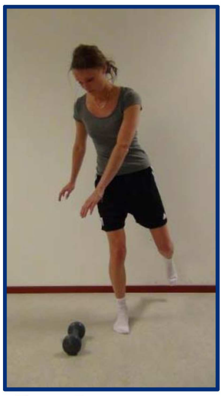

Figure 4

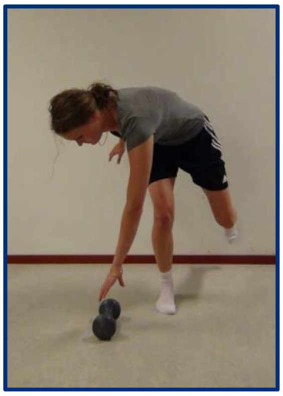

Figure 5

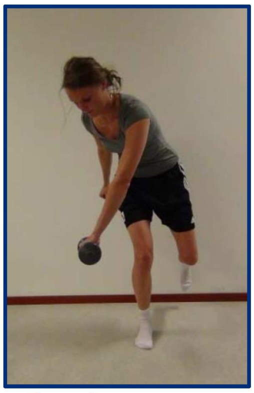

Figure 6

van der Graaff SJA, *et al. Br J Sports Med* 2022; 56:870–876. doi: 10.1136/bjsports-2021-105059

Supplementary Figure 1. Estimated IKDC score* for as treated analyses per measurement period

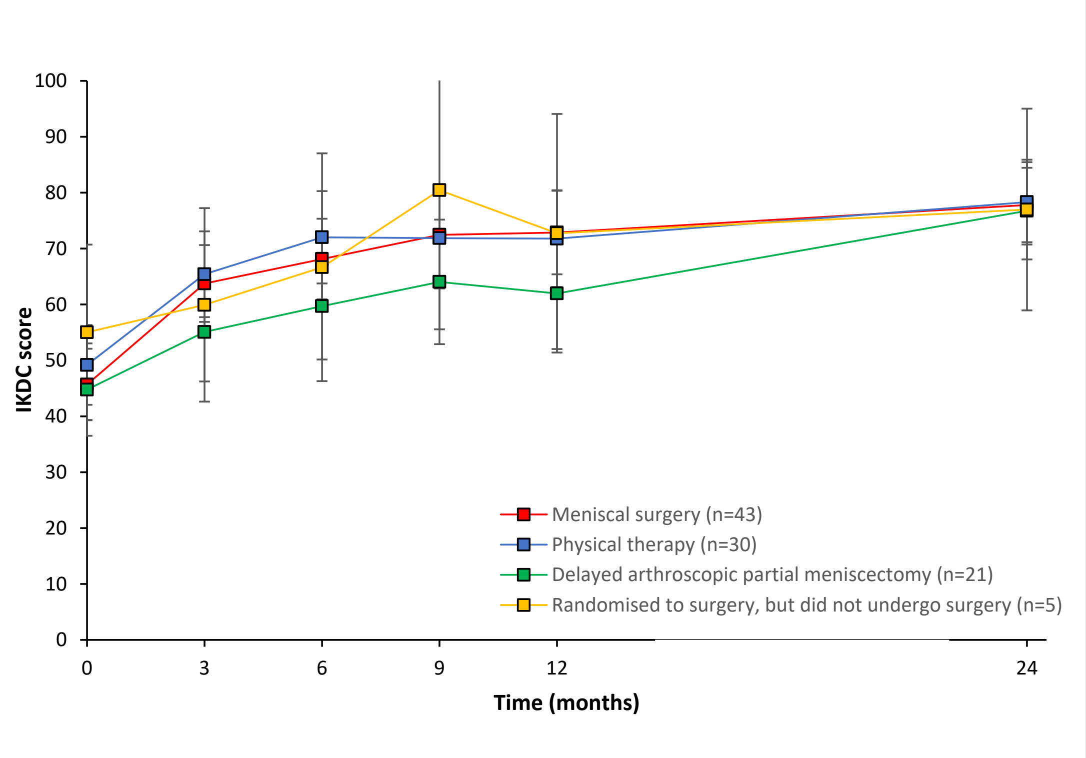

	baseline	3 months	6 months	9 months	12 months	24 months
Meniscal surgery (n=43)	46 (39 to 52)	64 (57 to 71)	68 (61 to 75)	72 (64 to 81)	73 (65 to 80)	78 (71 to 84)
Physical therapy (n=30)	49 (42 to 56)	65 (58 to 73)	72 (64 to 80)	72 (63 to 81)	72 (63 to 80)	78 (71 to 86)
Delayed arthroscopic partial menisectomy (n=21)	45 (37 to 53)	55 (46 to 64)	60 (50 to 69)	64 (53 to 75)	62 (52 to 72)	77 (68 to 85)
Randomised to surgery, but did not undergo surgery (n=5)	55 (39 to 71)	60 (43 to 77)	67 (46 to 87)	80 (56 to 105)	73 (51 to 94)	77 (59 to 95)
Primary outcome available, %	*98*	*77*	*70*	*63*	*75*	*91*

* adjusted for surgeon
Error bars represent 95% confidence intervals

Supplementary Table 1. Secondary outcomes* for as randomized analyses during follow-up

	Arthroscopic partial meniscectomy	Physical therapy	Between group difference
**KOOS baseline**			
pain	54.1 (48.2; 60.1)	59.5 (53.8; 65.2)	
symptoms	55.6 (49.7; 61.5)	62.9 (57.3; 68.5)	
ADL	61.3 (55.0; 67.6)	68.9 (62.9; 75.0)	
sport	30.2 (22.4; 38.0)	34.9 (27.4; 42.4)	
QoL	33.7 (28.0; 39.3)	35.4 (30.0; 40.8)	
**KOOS 24 months**			
pain	85.7 (79.0; 92.4)	83.8 (77.4; 90.2)	1.9 (-5.7; 9.6)
symptoms	81.8 (75.4; 88.2)	81.4 (75.2; 87.5)	0.5 (-6.6; 7.5)
ADL	92.0 (86.6; 97.5)	89.2 (84.1; 94.4)	2.8 (-3.3; 8.9)
sport	70.1 (60.5; 79.7)	69.3 (60.1; 78.6)	0.8 (-12.5; 14.0)
QoL	67.2 (59.1; 75.3)	65.8 (58.0; 73.6)	1.4 (-9.3; 12.0)
**NRS rest**			
baseline	3.9 (3.1; 4.6)	2.9 (2.2; 3.7)	
3 months	1.8 (1.1; 2.5)	1.6 (0.9; 2.2)	0.3 (-0.5; 1.0)
6 months	2.0 (1.2; 2.8)	1.3 (0.6; 2.1)	0.6 (-0.3; 1.6)
9 months	1.8 (0.8; 2.7)	1.5 (0.7; 2.4)	0.2 (-0.8; 1.3)
12 months	1.5 (0.6; 2.4)	1.7 (0.9; 2.6)	-0.2 (-1.2; 0.8)
24 months	1.2 (0.4; 1.9)	1.2 (0.5; 2.0)	-0.1 (-0.8; 0.7)
**NRS activity**			
baseline	6.6 (5.9; 7.3)	6.2 (5.5; 6.8)	
3 months	4.0 (3.2; 4.8)	3.8 (3.1; 4.6)	0.1 (-0.9; 1.1)
6 months	3.6 (2.7; 4.6)	2.9 (2.0; 3.8)	0.8 (-0.5; 2.0)
9 months	2.8 (1.7; 3.8)	3.1 (2.1; 4.0)	-0.3 (-1.7; 1.1)
12 months	2.4 (1.4; 3.3)	3.4 (2.5; 4.3)	-1.0 (-2.2; 0.2)

van der Graaff SJA, *et al. Br J Sports Med* 2022; 56:870–876. doi: 10.1136/bjsports-2021-105059

24 months	2.8 (1.9; 3.7)	2.4 (1.5; 3.3)	0.4 (-0.8; 1.5)
**Lysholm**			
baseline	66.9 (61.3; 72.5)	70.0 (64.7; 75.3)	
12 months	88.0 (82.3; 93.7)	83.0 (77.5; 88.4)	5.0 (-1.9; 11.9)
24 months	89.4 (84.8; 93.9)	88.3 (83.6; 93.1)	-1.0 (-6.2; 4.1)
**WOMET**			
baseline	38.1 (31.9; 44.4)	42.6 (36.6; 48.5)	
3 months	59.8 (51.9; 67.6)	59.4 (52.0; 66.8)	0.4 (-8.7; 9.5)
6 months	62.7 (54.8; 70.7)	65.8 (58.2; 73.4)	-3.1 (-12.4; 6.2)
9 months	71.9 (63.2; 80.7)	66.2 (58.2; 74.3)	5.7 (-4.8; 16.3)
12 months	70.8 (62.3; 79.3)	65.3 (57.1; 73.5)	5.5 (-4.9; 16.0)
24 months	71.9 (63.5; 80.2)	75.6 (67.6; 83.7)	-3.8 (-13.8; 6.2)
**Tegner****			
baseline	6.5 (5.9; 7.1)	6.4 (5.8; 7.0)	
3 months	3.8 (3.2; 4.5)	3.8 (3.2; 4.4)	0.0 (-0.8; 0.9)
6 months	4.6 (3.9; 5.4)	4.1 (3.4; 4.8)	0.6 (-0.4; 1.6)
9 months	5.0 (4.2; 5.8)	5.3 (4.5; 6.1)	-0.3 (-1.4; 0.8)
12 months	5.5 (4.8; 6.3)	4.4 (3.6; 5.1)	1.1 (0.1; 2.2)
24 months	5.4 (4.7; 6.1)	5.0 (4.4; 5.7)	0.3 (-0.6; 1.3)
**Satisfaction with knee function**			
baseline			
24 months	71.6 (63.7; 79.6)	70.1 (62.4; 77.8)	1.5 (-9.3; 12.3)

Data is presented as mean and 95% confidence interval between parentheses.
* adjusted for surgeon
** Tegner baseline score is pre-trauma score
Percentage of outcome available per time point: baseline 98%, 3 months 77%, 6 months 70%, 9 months 63%, 12 months 75%, 24 months 91%.
